# Dendritic nonlinearities enable PFC microcircuits to serve as predictive modules of persistent activity

**DOI:** 10.1186/1471-2202-14-S1-P319

**Published:** 2013-07-08

**Authors:** Athanasia Papoutsi, Panagiotis C Petrantonakis, Panayiota Poirazi

**Affiliations:** 1 Institute of Molecular Biology and Biotechnology, Foundation for Research and Technology, Heraklion, Crete, 70013, Greece; 2 Department of Biology, University of Crete, Heraklion, Crete, 71409, Greece

## 

The ability to monitor and probe the activity of large neuronal networks both *in vivo *and *in vitro *has recently established that neurons of various brain regions are organized into spatially restricted clusters (or small assemblies) that are bi-directionally connected, share common inputs and are co-activated during behavioral tasks [1,2]. Investigations regarding the functional implications of such neuronal clustering have proposed that this modularity may underlie the spiking irregularities seen in cortical activity *in vivo *[3] or code for the execution of a voluntary movement [4]. In the prefrontal cortex (PFC), such microcircuits are proposed to support the spontaneous emergence of Up and Down states [5], a phenomenon linked to persistent activity, which is the cellular correlate of working memory.

In this work we investigate the functional role of PFC microcircuits in the expression of persistent activity, focusing on the contribution of nonlinear dendritic properties to the induction, termination, and coding of upcoming state transitions.

Towards this goal we developed a layer V PFC microcircuit consisting of 7 pyramidal neurons and 2 interneurons implemented in the NEURON simulation environment. Modelling equations for the biophysical mechanisms used have been reported in [6,7]. All neuron models were biophysically detailed but morphologically simplified and were validated regarding their intrinsic, synaptic and connectivity properties (e.g. number of synapses, latencies etc). Our results show that the non-linear integration of synaptic inputs at the basal dendrites of pyramidal neurons, mediated by the induction of NMDA-spikes, is imperative for the emergence of the persistent state in the microcircuit, but this necessity disappears when increasing the network size. Moreover dendritic versus somatic specific alterations of ionic currents (such as the R type VGCCs) differentially modulate persistent activity induction, substantiating the critical role of location specific effects of various neuromodulators. Finally, we find that several features of the network activity *prior *to the induction and/or termination of persistent firing contain predictive information of the upcoming state-transition(s), which is readily available to downstream neurons. These findings suggest that PFC microcircuits may serve as tunable and predictive modules of persistent activity and subsequently working memory.
